# Supplementary material for: Experimental study of thermal rectification in suspended monolayer graphene
Source: Nat Commun. 2017 Jun 13;8:15843. doi: 10.1038/ncomms15843 (PMC5474737; doi:10.1038/ncomms15843)
Supplement: Supplementary Information — Supplementary Figures and Supplementary Tables. [file ncomms15843-s1.pdf]

Supplementary Note 1 Raman measurement of suspended monolayer graphene

We have performed Raman measurement on the suspended monolayer graphene sample for testing the lattice quality.

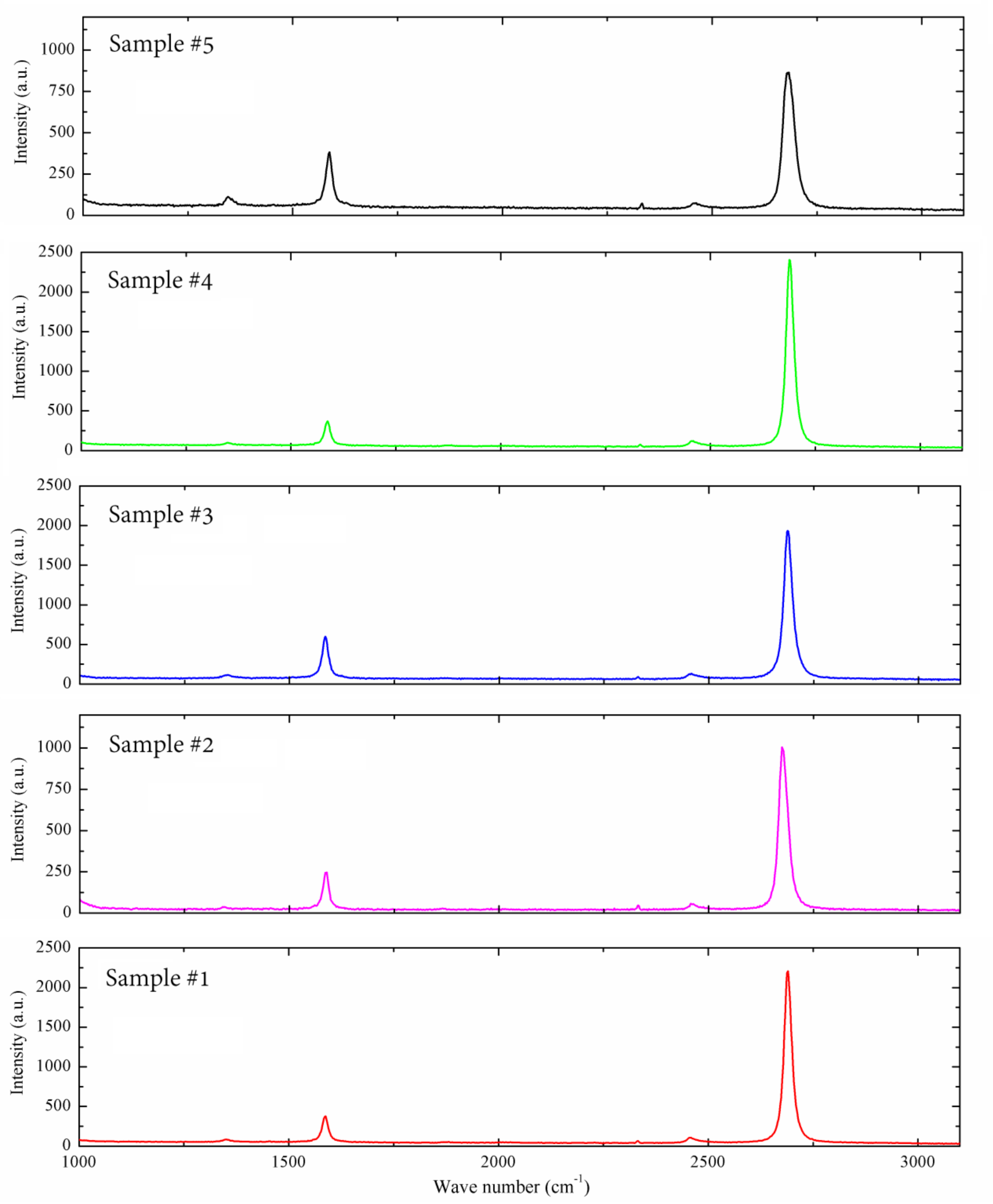

Supplementary Figure 1 Raman spectra of five suspended monolayer graphene

samples. The Raman measurement was performed for the suspended monolayer graphene samples. 532 nm excitation laser was used in the experiment and the exposure time was set as 90s. The monolayer graphene #5 was tested after cutting the rest monolayer graphene ribbon by low dose FIB (the radiation time was less than 1s). It is noted that the D-band peak of graphene sample #5 was a little higher than the other samples.

Supplementary Figure 1 displays the measured Raman spectra of five suspended monolayer graphene samples. The measurement was conducted by using Nano Photon^{TM} Raman spectrometer with 532 nm excitation laser. The large 2D band/ G band ratio and negligible D band peak indicate the monolayer structure and good sample quality. As shown in Fig. 5 of the main article, the rest graphene ribbon connected with the sample #5 was cut off by low dose FIB. The ion beam was focused away from the sample and the radiation time was less than 1s. The D-band peak of graphene sample #5 was a little higher than the other samples. However, such small damage to the graphene lattice had no significant effect on the thermal conductivity. The measured thermal conductivity of sample #5 was the same as the values of the other pristine samples. In fact, the total ion beam dose played an important role in reducing the thermal

conductivity of graphene. In order to create nanopores in the samples #1, #2 and #3, an ion beam was directly focused on graphene and the radiation time was long (~10s). As a result, the thermal conductivity of modified graphene decreased significantly.

Supplementary Note 2 Measurement uncertainty analysis

Before thermal measurement, both sensors connected with the monolayer graphene were annealed by large bias current heating for about 1 hour. The electrical resistance of sensor decreased during the annealing process. We monitored the resistance change of sensor and stopped the annealing until the resistance reached a stable value. The resistance of sensor was reduced by ~4% after annealing.

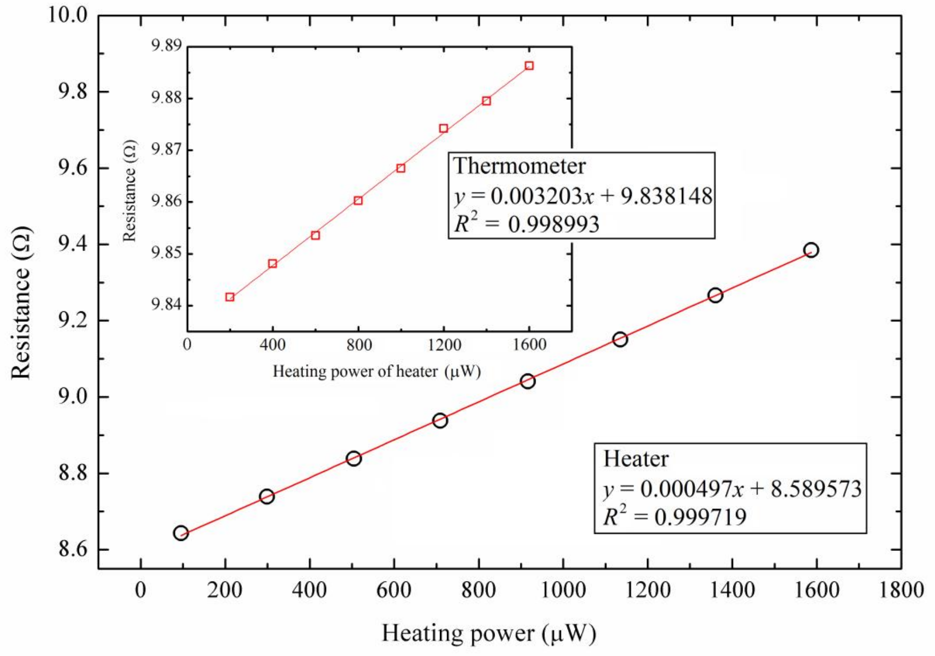

Supplementary Figure 2 Resistance changes of two sensors connected with pristine graphene sample #1. Two sensors were separately used as a heater and a thermometer.

The inset shows the resistance change of thermometer as a function of heating power of heater. The linear regression results are displayed in the figure.

In the H-type sensor method, one sensor was used as an electrical heater while the other sensor was used as a precise thermometer. By reversing the role of heater and thermometer, the heat flow direction through the graphene sample could be simply changed. Supplementary Figure 2 shows the original experimental data of graphene sample #1 before FIB modification. The resistance change reflected the average temperature change of the sensor. The maximum temperature rise of heater was controlled at ~35 K. We chose such high temperature rise in order to achieve a temperature response more than 0.2 K (in the case of the modified graphene sample with the lowest thermal conductivity) at the thermometer. A high signal-noise ratio was obtained at 0.2 K temperature rise and favorable to confirm the thermal rectification behavior. The resistance-temperature relationship was calibrated for each sensor at different ambient temperatures. In Supplementary Figure 2, the resistance change of thermometer was 0.0447 Ω and the corresponding temperature change was 1.90 K.

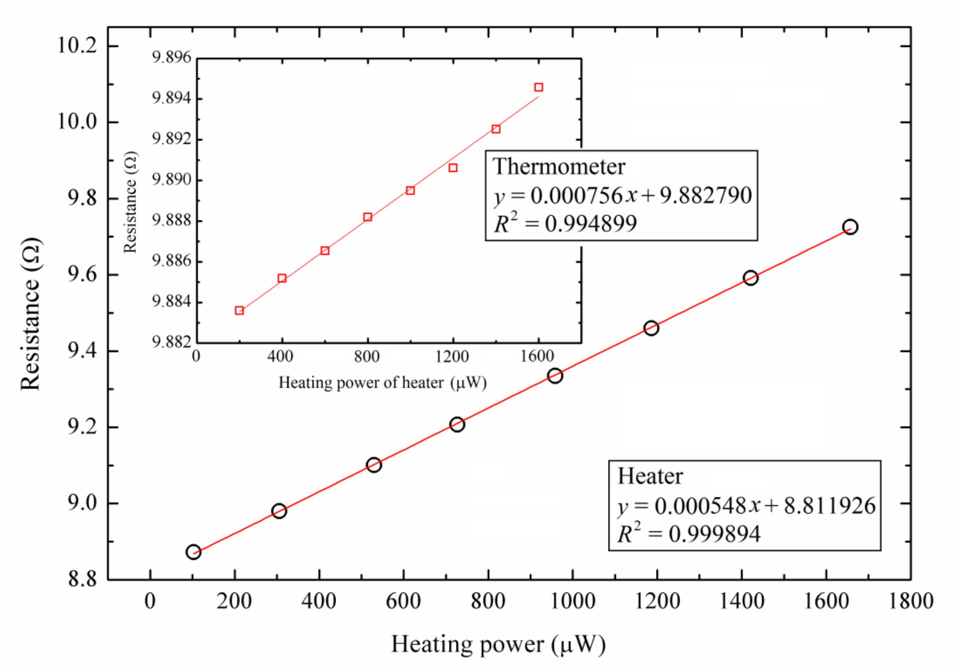

Supplementary Figure 3 Resistance changes of two sensors connected with the modified graphene sample #1. The thermal conductivity of modified graphene was much smaller than the value of pristine sample. As a result, the resistance change (temperature change) of the thermometer was much smaller than the result in Supplementary Figure 2 under the same heating power condition.

Supplementary Figure 3 shows the result of the same graphene sample after defect-engineering. Some nanopores were created in the bottom of sample by using FIB. At the same temperature rise of the heater, the resistance change of thermometer was 0.0110 Ω and the corresponding temperature change was 0.47 K, much smaller than the result in Supplementary Figure 2. In the experiment, the detectable minimum resistance

change of sensor was 0.0001 Ω, which corresponded to a temperature resolution of 0.01 K.

Based on a heat conduction model of H-type sensor, the heat flow Q through the suspended monolayer graphene can be estimated as:

$$Q = P\frac{\Delta T_{\mathrm{t}}}{\Delta T_{\mathrm{h}} + \Delta T_{\mathrm{t}}}, \qquad \text{(Supplementary Equation 1)}$$

where P is the heat generation in the sensor as a heater, ΔT_{h} and ΔT_{t} are the temperature rises of heater and thermometer, respectively. The heat generation P is determined as:

$$P = I_{\mathrm{h}}^2 R_{\mathrm{h}}, \qquad \text{(Supplementary Equation 2)}$$

where I_{h} and R_{h} are the current and resistance of heater, respectively. Based on a one-dimensional heat conduction model, the thermal conductivity λ of graphene can be estimated as:

$$\lambda = \frac{QL}{\left(\Delta T_{\mathrm{h}} - \Delta T_{\mathrm{t}}\right)}\frac{1}{dW}, \qquad \text{(Supplementary Equation 3)}$$

where L, W and d are the length, width and thickness of the graphene sample, respectively. Then, the error propagation rule gives:

$$\left(\frac{\delta\lambda}{\lambda}\right)^2 = \left(\frac{\delta Q}{Q}\right)^2 + \left(\frac{\delta\left(\Delta T_{\mathrm{h}} - \Delta T_{\mathrm{t}}\right)}{\Delta T_{\mathrm{h}} - \Delta T_{\mathrm{t}}}\right)^2 + \left(\frac{\delta L}{L}\right)^2 + \left(\frac{\delta W}{W}\right)^2 + \left(\frac{\delta d}{d}\right)^2, \qquad \text{(Supplementary Equation 4)}$$

$$\left(\frac{\delta Q}{Q}\right)^2 = 2\left(\frac{\delta I_{\mathrm{h}}}{I_{\mathrm{h}}}\right)^2 + \left(\frac{\delta R_{\mathrm{h}}}{R_{\mathrm{h}}}\right)^2 + \left(\frac{\delta\left(\Delta T_{\mathrm{t}}\right)}{\Delta T_{\mathrm{t}}}\right)^2 + \left(\frac{\delta\left(\Delta T_{\mathrm{h}} + \Delta T_{\mathrm{t}}\right)}{\Delta T_{\mathrm{h}} + \Delta T_{\mathrm{t}}}\right)^2 \qquad \text{(Supplementary Equation 5)}$$

The relative uncertainty of each parameter is determined as following:

The current I_h was measured by using a high precision digital multimeter (Keithley 2002) and a standard 100 Ω resistor (Yokogawa 2792A). The uncertainty was $\delta I_h / I_h <$ 0.004%. Using a four-probe method, the resistance R_h was measured as V_h / I_h. Thus, the uncertainty was $\frac{\delta R_h}{R_h} = \sqrt{\left(\frac{\delta V_h}{V_h}\right)^2 + \left(\frac{\delta I_h}{I_h}\right)^2} <$ 0.006%. The length and width of graphene were determined by the SEM image. The size of one pixel was translated to 15 nm in space. Thus, the uncertainties of L and W were $\delta L / L \sim \delta W / W \sim$ 0.5%. The thickness of monolayer graphene was 0.334 nm as recommended in Supplementary Reference 1. As described above, the average temperature rise of heater was ~35 K. The temperature resolution of sensor was 0.01 K. Thus, the uncertainty of ΔT_h was $\delta(\Delta T_h) / \Delta T_h \sim$ 0.03%. The largest measurement uncertainty came from the temperature rise of thermometer. For the pristine graphene sample with high thermal conductivity, average ΔT_t was approximately 2.1 K. Thus, the uncertainty was $\delta(\Delta T_t) / \Delta T_t \sim$ 0.5%. For the modified graphene after FIB radiation, the thermal conductivity was significantly reduced. As a result, ΔT_t was much smaller than that of the pristine sample. As discussed in the main article, thermal rectification was caused by the varied temperature dependence of thermal conductivity in the nanopore region and the region without defects. The thermal conductivity of monolayer graphene with nanopores was almost temperature independent. Therefore, the average thermal conductivity of modified

graphene was responsible for the final thermal rectification factor, whose uncertainty was decided by the average temperature rise. Average ΔT_t of the modified graphene samples was 0.4 K and the uncertainty was $\delta(\Delta T_t) / \Delta T_t$ ~ 2.3%.

Substituting all these uncertainties into Eqs. (S4) and (S5), $\delta Q / Q$ and $\delta\lambda / \lambda$ are decided as 2.4% and 2.5%, respectively. On the other hand, the temperature fluctuation of Peltier stage was ~ 0.04 K. The uncertainty of two-dimensional thermal analysis model (in part 4) was smaller than 2%. Taking all these factors into account, the final uncertainty of thermal conductivity measurement was less than 5%.

Meanwhile, we also estimated the influence caused by the heat loss through the thermal radiation from the sensor to the environment in vacuum. The largest temperature difference between the sensor and environment was about 50 K. Thus, the maximum heat loss through thermal radiation was calculated as $J = \varepsilon\sigma A(T_s^4 - T_0^4) = 0.025\times5.67\times10^{-8}\times2.4\times10^{-11}\times(350^4 - 300^4) = 0.00023$ μW, where J, ε, σ, A, T_s and T_0 are the heat loss energy, emissivity coefficient of gold, Stefan–Boltzmann constant, surface area of sensor, temperatures of sensor and environment, respectively. In comparison, the minimum electrical heating power of sensor in the experiment was about 95 μW, which was much larger than the heat loss energy. As a result, the thermal radiation can be safely neglected in the current study.

Supplementary Note 3 Impact of contact thermal resistance

In this work, the Au sensors were directly deposited on monolayer graphene/SiO_2/Si by an electron beam physical vapor deposition method. Then, the sensors and monolayer graphene were suspended by using a combined wet/gas etching technique [2]. If the graphene membrane was transferred onto the metal film, some residues or air might be trapped in between. Hence, the contact thermal resistance was supposed to be notably increased. In order to estimate the contact thermal resistance, a fin thermal resistance model was utilized [3]:

$$R_c = \left[\sqrt{\frac{\lambda A W}{R_{int}}} \tanh\left(\sqrt{\frac{W}{\lambda A R_{int}}} L_c \right) \right]^{-1}, \quad \text{(Supplementary Equation 6)}$$

where R_c is the contact thermal resistance, $R_{int} = 4 \times 10^{-8}$ m^2K/W is the interfacial thermal resistance per unit area [4], λ and W are the thermal conductivity and width of the graphene sample, $A = dW$ is the cross-sectional area (d is the thickness of monolayer graphene), L_c is the graphene-metal contact length. The calculated contact thermal resistances by Supplementary Equation 6 are summarized in Supplementary Tables 1 and 2.

Supplementary Table 1 Contact thermal resistances of pristine graphene samples

	L (μm)	W (μm)	L_c (μm)	λ_0 ($Wm^{-1}K^{-1}$)	$R_0 \times 10^6$ (KW^{-1})	$R_c \times 10^5$ (KW^{-1})	R_c / R_0 (-)
Sample #1	2.66	2.80	0.97	2174	1.29	1.58	0.123
Sample #2	2.76	3.16	1.00	2234	1.15	1.40	0.122
Sample #3	2.93	3.21	1.04	2400	1.12	1.38	0.123
Sample #4	2.85	4.08	1.00	1845	1.11	1.09	0.098
Sample #5	3.17	5.22	0.93	1918	0.93	0.85	0.091

Supplementary Table 2 Contact thermal resistances of modified graphene samples

	L (μm)	W (μm)	L_c (μm)	λ_0 ($Wm^{-1}K^{-1}$)	$R_0 \times 10^6$ (KW^{-1})	$R_c \times 10^5$ (KW^{-1})	R_c / R_0 (-)
Sample #1 after FIB	2.66	2.80	0.97	405	6.90	1.58	0.023
Sample #2 after FIB	2.76	3.16	1.00	443	5.80	1.40	0.024
Sample #3 after FIB	2.93	3.21	1.04	511	5.25	1.38	0.026

Supplementary Tables 1 and 2 display the contact thermal resistances of graphene samples before and after FIB modification. The average thermal conductivity from 280 K to 340 K was used for calculation. For the pristine graphene with high thermal conductivity, the contact thermal resistance was ~12% of the thermal resistance of monolayer graphene (R_0). After FIB radiation, the thermal conductivity of sample

decreased significantly and the ratio between R_c and R_0 was reduced to be less than 3%. The estimated contact thermal resistance was taken into account for determining the final thermal conductivity of sample.

The contact thermal resistance alone cannot explain the thermal rectification phenomenon observed in the experiment. For the rectangle graphene ribbons (#1 to #4), the contact thermal resistances at the two ends of graphene are the same because of the uniform width. We have measured the thermal conductivities of pristine graphene in two opposite heat flux directions before the defect engineering and carbon deposition. No thermal rectification was found in these rectangle graphene ribbons. Moreover, the contact resistance was constant during FIB or EBID since the electron beam or ion beam could not reach the monolayer graphene under the metal film sensor. For the trapezoid graphene ribbon #5, the contact thermal resistances are different at two ends because of the different widths. However, the contact thermal resistance is almost independent of temperature from 300K to 400K due to the experimental reports on the constant thermal conductivity of supported graphene [5] and the constant interfacial thermal conductance between graphene and metallic film near room temperature [4]. As a result, the contact thermal resistance has no influence on the thermal rectification.

Supplementary Note 4 Thermal analysis based on a finite element model

In order to determine the thermal conductivity of each graphene sample, we have conducted thermal analysis by using a commercial finite element software COMSOL Multiphysics^{TM}. The thermal conductivity of Au film was calibrated separately in the H-type sensor without graphene. The geometric sizes of monolayer graphene and sensor were measured in the SEM image. The Joule heating power of sensor was precisely measured in the experiment. In the thermal analysis model, thermal conductivity of monolayer graphene was the only unknown parameter.

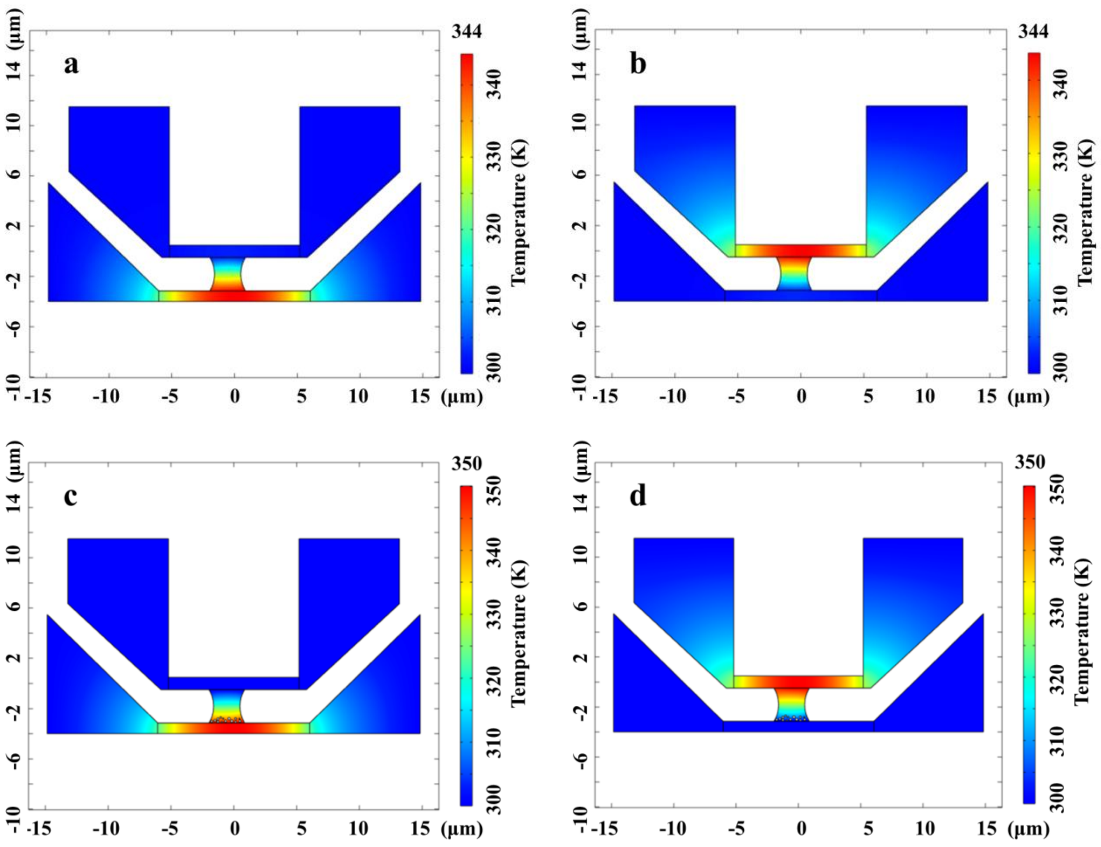

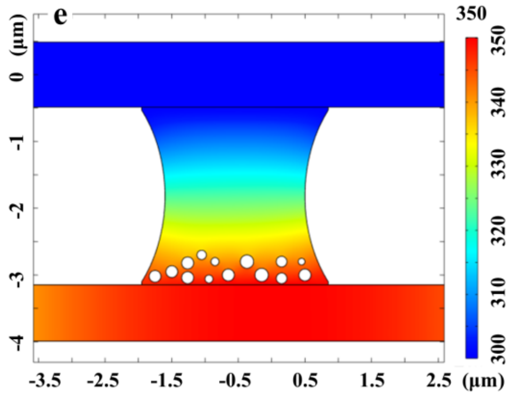

Supplementary Figure 4 Thermal analysis result of graphene sample #1. (a, b) Temperature distribution of pristine graphene in two opposite heat flux directions. (c, d) Temperature distribution of defect-engineered graphene. (e, f) Zoom-in of graphene part in the middle.

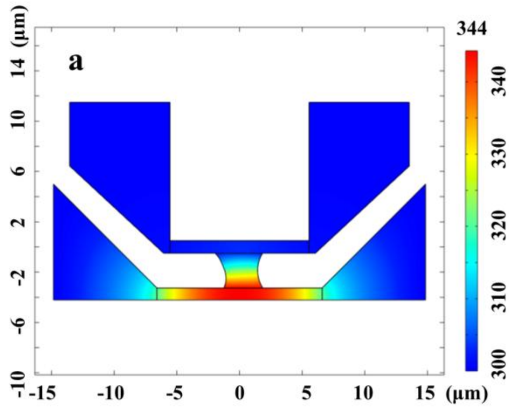

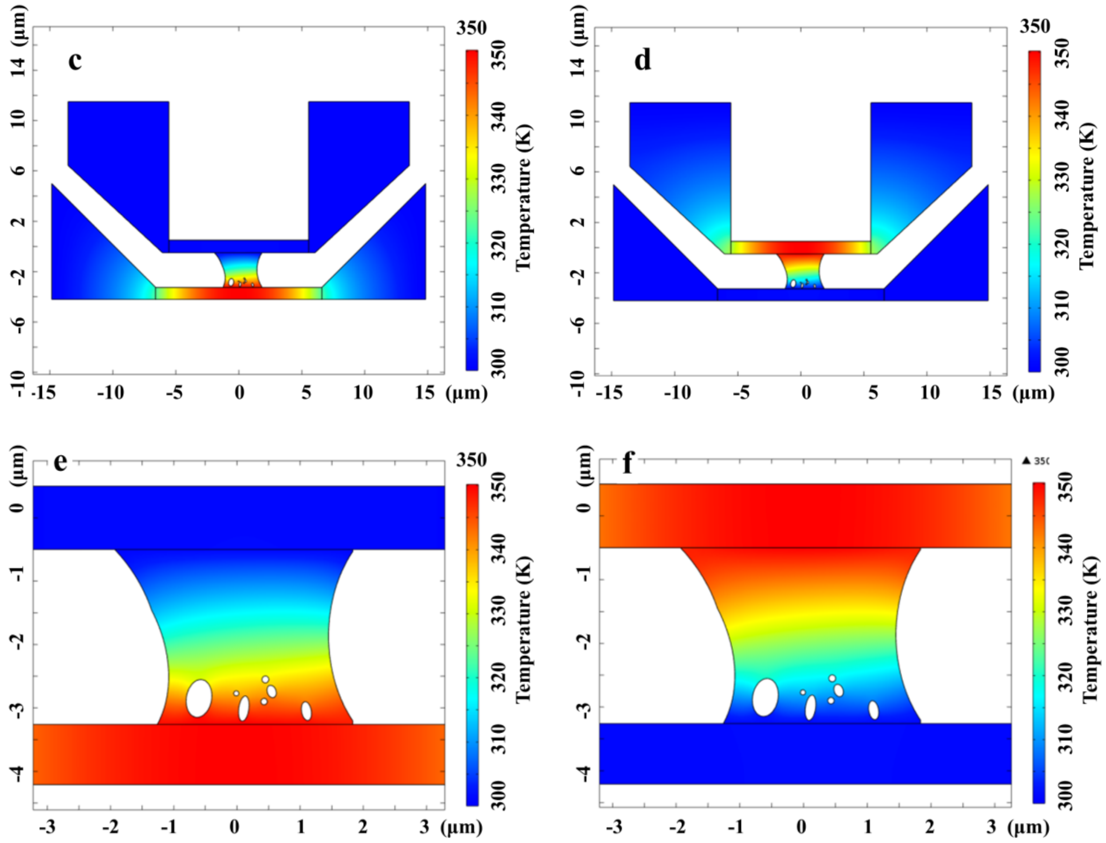

Supplementary Figure 5 Thermal analysis result of graphene sample #2. (a, b) Temperature distribution of pristine graphene in two opposite heat flux directions. (c, d) Temperature distribution of defect-engineered graphene. (e, f) Zoom-in of graphene part in the middle.

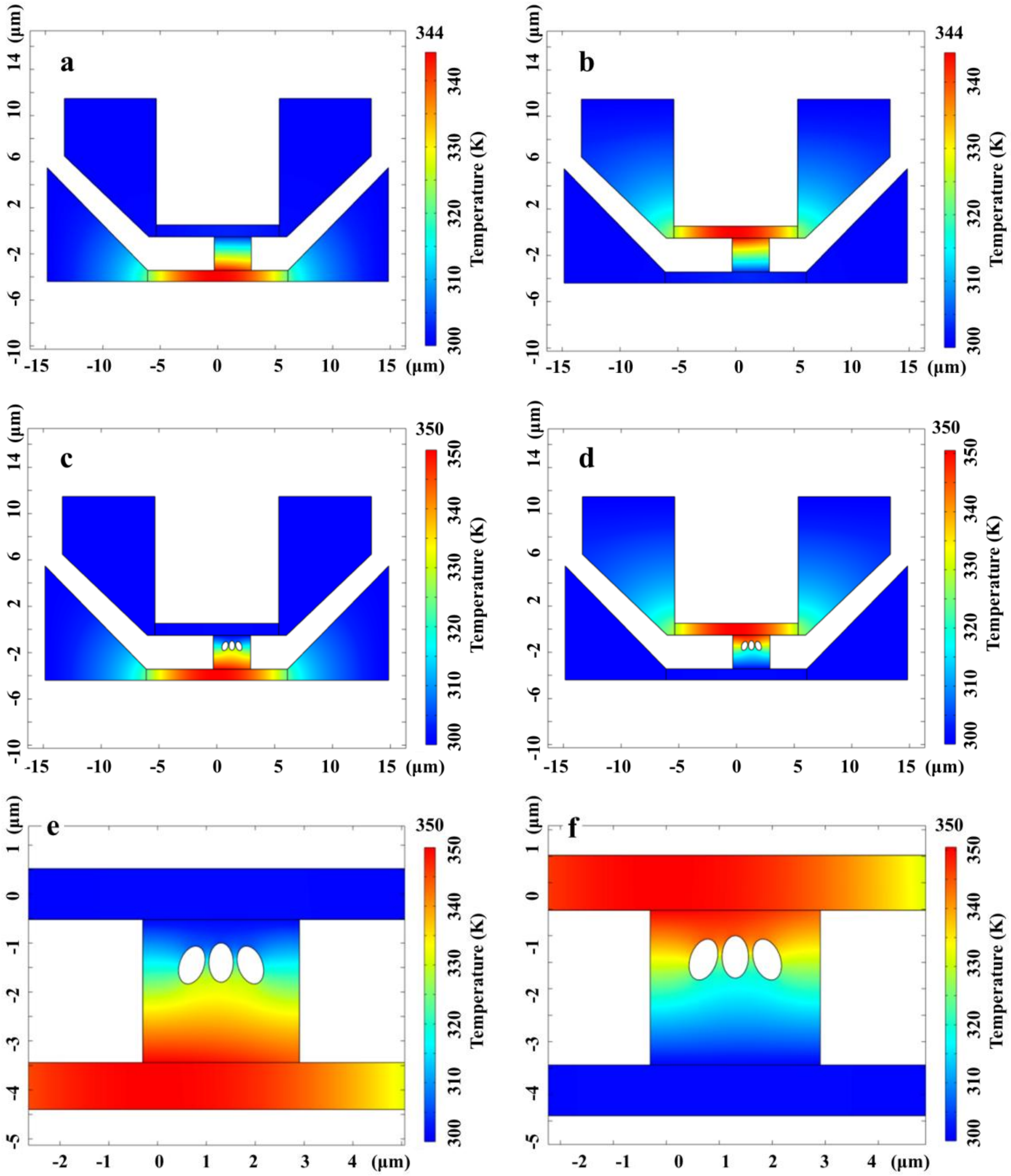

Supplementary Figure 6 Thermal analysis result of graphene sample #3. (a, b) Temperature distribution of pristine graphene in two opposite heat flux directions. (c, d) Temperature distribution of defect-engineered graphene. (e, f) Zoom-in of graphene part in the middle.

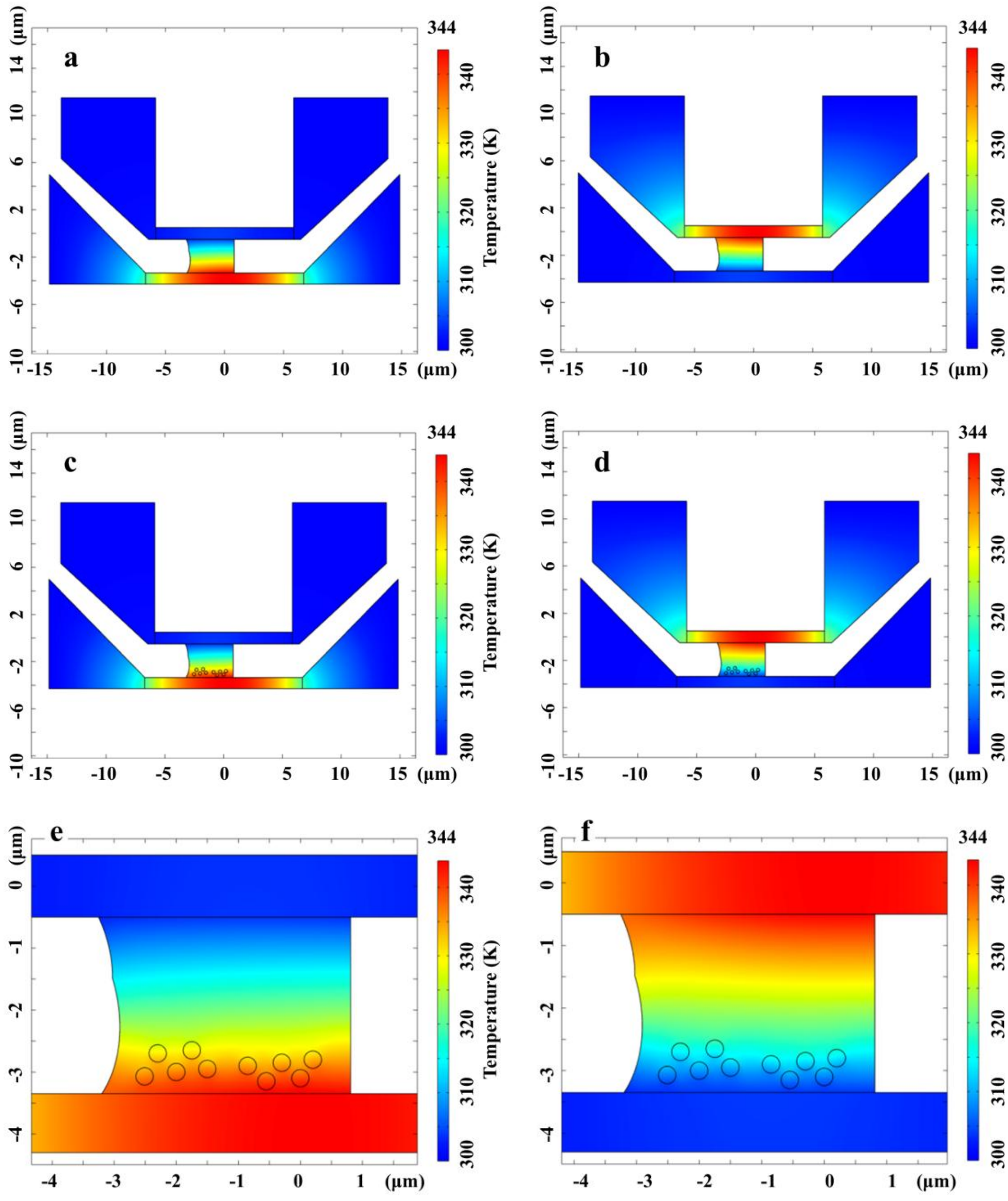

Supplementary Figure 7 Thermal analysis result of graphene sample #4. (a, b) Temperature distribution of pristine graphene in two opposite heat flux directions. (c, d) Temperature distribution of graphene with deposited nanoparticles. (e, f) Zoom-in of graphene part in the middle.

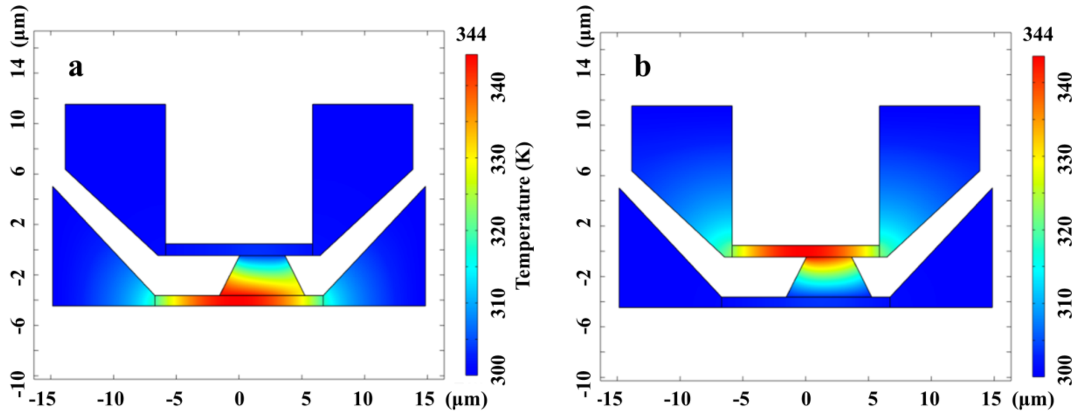

Supplementary Figure 8 Thermal analysis result of graphene sample #5. (a, b) Temperature distribution of graphene with a tapered width in two opposite heat flux directions.

The thermal analysis results are shown from Supplementary Figure 4 to Supplementary Figure 8, including nine models. Every model has two conditions, under which heat flows either from the upper sensor to the bottom sensor or vice versa. The size and location of nanopores or nanoparticles were decided based on the SEM image. It should be noted that the average temperature rise of sensor was kept the same when the heat flow direction was reversed. Thus, the average temperature of graphene sample was constant in either heat flow direction. In this way, the observed thermal rectification phenomenon could be confirmed to be induced by the asymmetric graphene nanostructures alone, not the temperature change of the sample.

Supplementary Methods 5 FIB/EBID apparatus

The nanomanufacturing and thermal measurement of monolayer graphene sample were performed by using FEI Versa 3D^{TM} dual-beam system. The prepared pristine monolayer graphene was placed on a Peltier stage inside the high-vacuum chamber and measured on site. Then, the FIB or EBID was used to create asymmetric structures in the graphene. Subsequently, the thermal conductivity was measured again without taking out the sample.

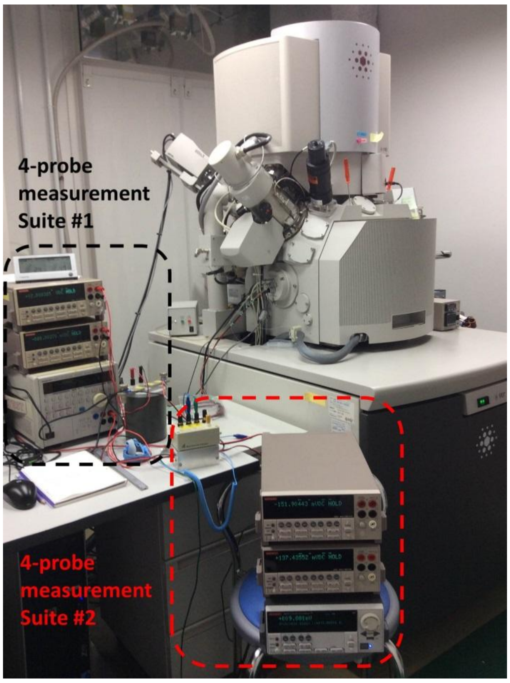

Supplementary Figure 9 Experimental apparatus for H-type sensor measurement. There are two measurement suites (two multimeters, DC-power supply and standard resistor) shown in the black and red dashed-line squares. They were used separately for measuring the electrical resistance of each sensor by 4-probe method. The FEI

dual-beam system was placed in the backside.

Supplementary Figure 9 shows the experimental apparatus for H-type sensor measurement. Two measurement suites were used for measuring the electrical resistances of two sensors separately. In each 4-probe measurement suite, there are two high-precision digital multimeters (Keithley 2002), a voltage/current source (Advantest R6243) and a standard 100 Ω resistor (Yokogawa 2792A). One advantage of this measurement system is that the heat flow direction can be easily reversed. One only needs to adjust the Joule heating power of two sensors to reverse the temperature gradient.

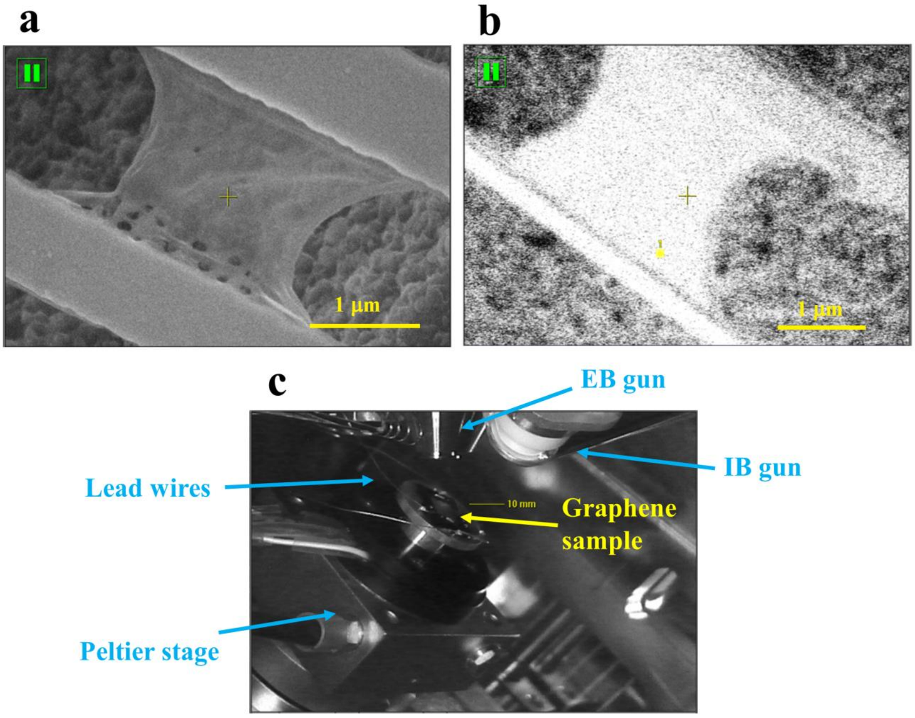

Supplementary Figure 10 FIB operation for drilling nanopores in graphene sample #1. (a) SEM image of graphene with nanopores. (b) image of graphene under ion beam mode. (c) Photo of electron beam (EB) gun, ion beam (IB) gun and graphene sample inside the vacuum chamber.

Supplementary Figure 10 shows how to operate FIB for creating nanopores in the suspended monolayer graphene. In the FIB image (b), the size and location of nanopores were designed. After FIB radiation, the SEM image (a) was used to observe and check the result.

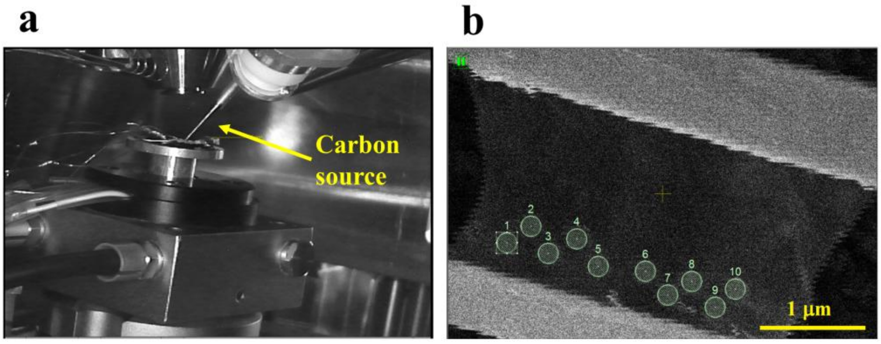

Supplementary Figure 11 EBID operation for depositing particles on graphene sample #4. (a) Photo of graphene sample on the Peltier stage. A carbon source needle is injected close to the sample surface. (b) SEM image for designing the size and location of nanoparticles on the graphene surface.

Supplementary Figure 11 shows how to deposit carbon nanoparticles on the monolayer graphene by using EBID. Before deposition, one needs to design the size and location of nanoparticles. In order to minimize the damage to the graphene lattice caused by electron beam radiation, we chose the lowest accelerating voltage (10 kV) for EBID. The electron beam current was 41 pA. As discussed in the main article, the thermal conductivity of monolayer graphene #4 after EBID was slightly reduced comparing with the result of the pristine sample. It demonstrates that the EBID process did not cause significant damage to the graphene lattice.

Supplementary Note 6 Lattice dynamics model for theoretical analysis

Theoretically predicted thermal conductivity of monolayer graphene is shown in Figs. 3 and 6 in the main article. The calculation was conducted based on a lattice dynamics model of phonon transport in monolayer graphene [6]:

$$\lambda = \frac{1}{4\pi k_{\mathrm{B}} T^2 \delta} \sum_{\substack{s=\mathrm{TA,LA,ZA,}\\ \mathrm{TO,LO,ZO}}} \int_{q_{\min}}^{q_{\max}} \left\{ \left[\hbar\omega_{\mathrm{s}}(q) v_{\mathrm{s}}(q)\right]^2 \times \tau_{\mathrm{s}}(q) \frac{\exp\left[\hbar\omega_{\mathrm{s}}(q)/k_{\mathrm{B}}T\right]}{\left(\exp\left[\hbar\omega_{\mathrm{s}}(q)/k_{\mathrm{B}}T\right]-1\right)^2} q \right\} \mathrm{d}q$$

(Supplementary Equation 7)

where k_{B}, $\hbar$, ω_{s}, τ_{s}, q and T are the Boltzmann constant, reduced Planck constant, phonon frequency, relaxation time, wave vector and temperature, respectively. δ is the interplanar spacing of graphite. $v_{\mathrm{s}} = \mathrm{d}\omega_{\mathrm{s}} /\mathrm{d}q$ is the group velocity. The subscript s stands

for six different phonon polarization branches, including three acoustic branches (TA, LA, ZA) and three optical branches (TO, LO, ZO). The relaxation time τ_s is calculated based on Matthiessen's Rule:

$$\tau_s(q)=\left[\frac{1}{\tau_{U,s}(q)}+\frac{1}{\tau_{B,s}(q)}+\frac{1}{\tau_{D,s}(q)}\right]^{-1}, \qquad \text{(Supplementary Equation 8)}$$

where $\tau_{U,s}$, $\tau_{B,s}$ and $\tau_{D,s}$ [7] are the relaxation times of phonon Umklapp scattering, boundary scattering and defect scattering, respectively.

$$\tau_{U,s}(q)=\frac{1}{\gamma_s^2}\frac{M\bar{v}_s^2}{k_B T}\frac{\omega_{s,max}}{\omega_s^2(q)}, \qquad \text{(Supplementary Equation 9)}$$

$$\tau_{B,s}(q)=\frac{W}{v_s(q)}\frac{1+p}{1-p}, \qquad \text{(Supplementary Equation 10)}$$

$$\tau_{D,s}(q)=\frac{G\sigma}{9\delta}\frac{v_s^3(q)}{4\pi^3 w_s^4(q)} \qquad \text{(Supplementary Equation 11)}$$

where γ_s, $\bar{v}_s$ and M are the Gruneisen parameter, average phonon velocity and mass of a graphene unit cell, respectively. $\omega_{s,max}$ and p are the maximum cut-off frequency and specularity parameter, respectively. G is the number of atoms per defect. $\sigma=4/(\sqrt{3}a^2)$ is the surface density of carbon atoms, where a = 2.49 Å is the graphene lattice constant.

Based on this model, three important phonon scattering mechanisms are estimated. For the pristine monolayer graphene before defect-engineering, the boundary scattering and Umklapp scattering play important roles. The mean free path of monolayer graphene is comparable to the width of sample at room temperature. Thus, the finite

width of graphene suppresses the thermal conductivity by phonon confinement at the lateral boundaries. The width dependence of thermal conductivity can be evaluated by the relaxation time τ_B. Meanwhile, the temperature dependence of thermal conductivity can be evaluated by the relaxation time τ_U. Higher temperature indicates stronger Umklapp scattering. Thus, τ_U is inversely proportional to temperature. Thermal conductivity of pristine monolayer graphene decreases with increasing temperature.

For the graphene with nanopores, the defect scattering dominates over the other two scattering mechanisms. τ_D is much smaller than τ_B and τ_U. The thermal conductivity of graphene is mainly influenced by the defect density, not the geometric size or temperature. The defect density was determined by the ion beam dose. Thus, long time radiation or high current ion beam will reduce the thermal conductivity significantly. As discussed in the main article, the thermal conductivity of defect-engineered monolayer graphene became almost temperature independent. The asymmetric distribution of defect density causes varied dependence of thermal conductivity on temperature and space. On this basis, one can design different monolayer graphene thermal rectifiers by introducing various asymmetric structural defects.

Supplementary Note 7 Molecular dynamics simulations

More details about the MD simulation are introduced in this section. To introduce the

temperature difference, the carbon atoms at two ends (six layers) were coupled with Nosé-Hoover heat bath at temperature T_L and T_R, (Fig. 7a), respectively. In our simulations, the average temperature (T_0) was set as 300 K and the values of temperature difference ($\Delta T = |T_L - T_R|$) were varied from 30 K to 200 K. As shown in Fig. 7a, J_+ (J_-) denotes the heat flux in the direction with the temperature bias $T_L > T_R$ ($T_L < T_R$). After the system reached the non-equilibrium steady state, the temperature and heat flux were averaged over 1 ns of non-equilibrium molecular dynamics (NEMD) run. The length of the trapezoid graphene was set as L. The width of the wide (narrow) end was set as W_1 (W_2). The pitch angle was set as θ. In the smallest model, we set L = 17 nm, W_1 = 22 nm, W_2 = 2 nm and θ = 30 °, similar to the geometric shape of sample #5.

Due to the unique two-dimensional nature of the trapezoid graphene sheet, the number of atoms grows tremendously as the length (L) and the width (W) increases. In the smallest model with 22 nm width, there were 7,420 C atoms. Then, we fixed the angle θ in the trapezoid (Fig. 7a in main article), and increased both L and W proportionally. The W_1 in the MD simulation system was increased to 200 nm and 440 nm. The total number of C atoms in the biggest graphene sheet was 2,985,734. To simulate such large system for 5 ns, each MD task required 52,560 CPU hours, which corresponded to a non-stop calculation time of 20 days on 120 CPUs. If the width of

graphene sheet further increases to 1 μm, it needs more than 2 months non-stop calculation. Due to the limited computational resources and time, it was not feasible for us to complete the MD simulation for micrometer-sized graphene sheet.

To simulate the carbon nanoparticle deposition, we used the heavy atoms (3 times of the atomic mass of carbon) to mimic the nanoparticles on sample #4. 400 heavy atoms were randomly distributed on the left part of the graphene sheet (Fig. 8a). The length and width of the asymmetrically deposited graphene were set as L and W, respectively. The length of the deposited nanoparticles region was set as L_R. The distance in the z-direction between the heavy atoms and pristine graphene was denoted as d. We set in our simulation that L = 17 nm, W = 5 nm, L_R =4 nm and d = 0.335 nm. The interaction between the heavy atoms and pristine graphene was described by a Lennard-Jones (LJ) potential:

$$V\left(r_{ij}\right)=4\varepsilon\left[\left(\sigma_{ij}/r_{ij}\right)^{12}-\left(\sigma_{ij}/r_{ij}\right)^{6}\right], \quad \text{(Supplementary Equation 12)}$$

In our model, the deposited atoms were frozen to mimic the deposited nanoparticles on graphene. We set ε = 0.3 eV and σ = 3.415 Å for the results shown in Fig. 8. We have performed additional simulations (Supplementary Figure 12) to test different LJ parameters and initial distance d. The result shows that the different parameters affect the energy distribution quantitatively, but the underlying mechanism for thermal

rectification is the same as that shown in Fig. 8 of the main article.

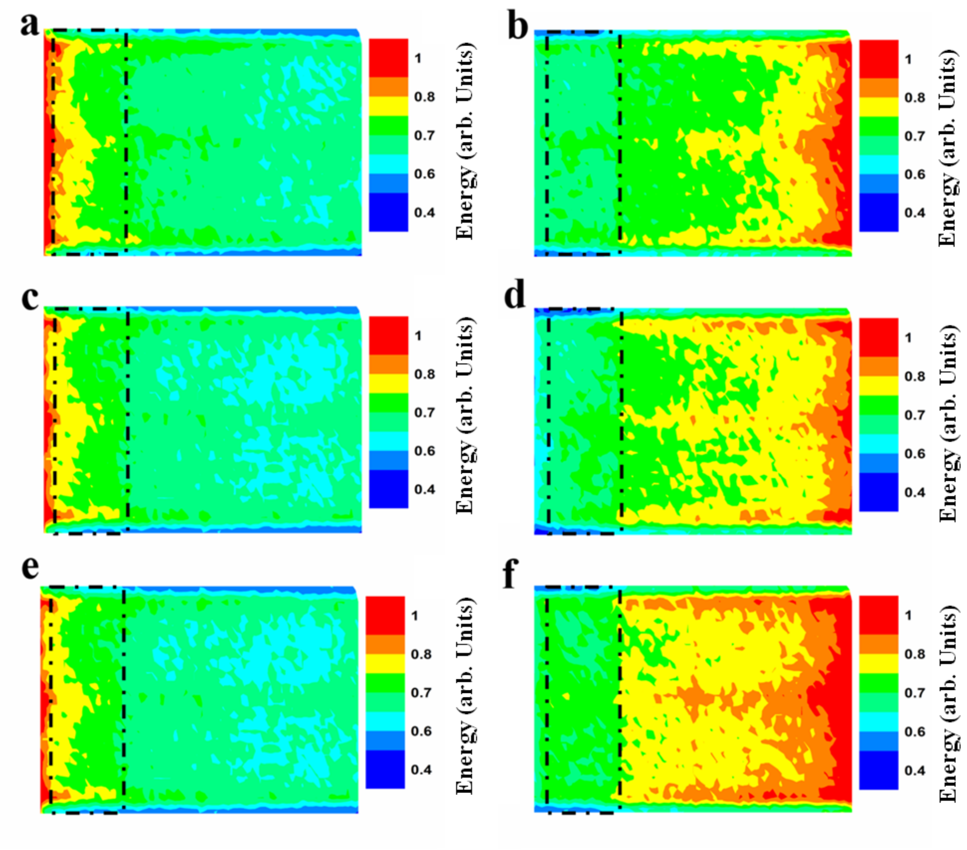

Supplementary Figure 12 The spatial energy distribution for the propagating phonon modes (P_λ >0.4) in the asymmetrically deposited graphene. (a, b) ε = 0.03 eV, σ = 3.415 Å and d = 0.335 nm. (c, d) ε = 0.15 eV, σ = 3.415 Å and d = 0.335 nm. (e, f) ε = 0.15 eV, σ = 3.415 Å and d = 0.67 nm. The dashed box denotes the deposited nanoparticles region.

In the lattice dynamics calculation, the eigen-frequency and eigen-vector for each

phonon mode in graphene were computed by using the GULP [8] package with the optimized Tersoff potential [9]. The same lattice structure shown in Fig. 7a and Fig. 8a was used in the lattice dynamics calculations, which contained 7420 C-atoms for the trapezoid graphene and 2800 C-atoms in the rectangular graphene. In the MD simulation, we set T_L = 400 K, T_R = 200 K and computed the steady-state local temperature of each atom as:

$$T_i = \frac{\langle m_i \mathbf{v}_i \cdot \mathbf{v}_i \rangle}{3k_B}, \qquad \text{(Supplementary Equation 13)}$$

where m_i and $\mathbf{v}_i$ are the mass and velocity of the ith atom, k_B is the Boltzmann constant, the angular bracket denotes the time average. This local temperature information was used in Supplementary Equation 13 to obtain the spatial energy distribution.

However, the lattice dynamics calculation was more strictly limited by the finite computational resources. The eigenvector and eigenfrequency was obtained by diagonalizing the dynamical matrix. For a unit cell of N atoms, the dynamical matrix scaled as $(3N)^2$. In the smallest model with 7,420 atoms, the size of dynamical matrix was already quite large. We attempted to double the graphene width (29,680 atoms in the unit cell) and repeat the lattice dynamics calculation on a computer cluster with a total memory of 512 GB. Unfortunately, the calculation on such huge dynamical matrix could not be executed due to the formidable memory requirement.

Supplementary References

[1] Pati, S. K., Enoki, T. & Rao, C. N. R. Graphene and its fascinating attributes. World Scientific Publishing Co. Pte. Ltd., Singapore (2011).

[2] Wang, H. D. et al. A general method of fabricating free-standing, monolayer graphene electronic device and its property characterization. *Sens. Actuator. A* **247,** 24-29 (2016).

[3] Mavrokefalos, A., Pettes, M. T., Zhou, F. & Shi, L. Four-probe measurements of the in-plane thermoelectric properties of nanofilms. *Rev. Sci. Instrum.* **78**, 034901 (2007).

[4] Koh, Y. K., Bae, M. H., Cahill, D. G. & Pop, E. Heat conduction across monolayer and few-layer graphenes. *Nano Lett.* **10**, 4363-4368 (2010).

[5] Seol, J. H. et al. Two-dimensional phonon transport in supported graphene. *Science* **328**, 213-216 (2010).

[6] Nika, D. L. & Balandin, A. A. Two-dimensional phonon transport in graphene. *J. Phys.: Condens. Matt.* **24,** 233203 (2012).

[7] Xie, G. F. et al. A bond-order theory on the phonon scattering by vacancies in two-dimensional materials. *Sci. Rep.* **4**, 05085 (2014).

[8] Gale, J. D. GULP: A computer program for the symmetry-adapted simulation of solids. *J. Chem. Soc., Faraday Trans.* **93**, 629-637 (1997).

[9] Lindsay, L. & Broido, D. A. Optimized Tersoff and Brenner empirical potential parameters for lattice dynamics and phonon thermal transport in carbon nanotubes and graphene. *Phys. Rev. B* **81,** 205441 (2010).
